# Flexural Behavior of Double-Skin Steel Tube Beams Filled with Fiber-Reinforced Cementitious Composite and Strengthened with CFRP Sheets

**DOI:** 10.3390/ma13143064

**Published:** 2020-07-09

**Authors:** Ahmed Al-Nini, Ehsan Nikbakht, Agusril Syamsir, Nasir Shafiq, Bashar S. Mohammed, Amin Al-Fakih, Waleed Al-Nini, Y. H. Mugahed Amran

**Affiliations:** 1 Civil and Environmental Engineering Department, Universiti Teknologi Petronas, Bandar Seri Iskandar 32610, Malaysia; ahmed_17005180@utp.edu.my (A.A.-N.); ehsan.nikbakht@utp.edu.my (E.N.); bashar.mohammed@utp.edu.my (B.S.M.); 2 Institute of Energy Infrastructure (IEI), Universiti Tenaga Nasional, Kajang 43000, Malaysia; 3 Department of Civil Engineering, Universiti Tenaga Nasional, Kajang 43000, Malaysia; 4 Almarja for Engineering and Cosultancies, 30th Street, Bait Bouse, Sana’a 452, Yemen; alnini.eng@gmail.com; 5 Faculty of Engineering, Thamar University, Dhamar 87246, Yemen; 6 Department of Civil Engineering, College of Engineering, Prince Sattam Bin Abdulaziz University, Alkharj 11942, Saudi Arabia; m.amran@psau.edu.sa; 7 Department of Civil Engineering, Faculty of Engineering and IT, Amran University, Amran 9677, Yemen

**Keywords:** HPCFDST beam, CFRP sheet, flexural stiffness, energy absorption, failure mode, moment capacity

## Abstract

The concrete-filled double skin steel tube (CFDST) is a more viable option compared to a concrete-filled steel tube (CFST) due to consisting a hollow section, while degradation is enhanced simply by using carbon fiber-reinforced polymer (CFRP). Hence, the stabilization of a concrete’s ductile strength needs high- performance fiber-reinforced cementitious conmposite. This study investigates the behavior of high-performance fiber-reinforced cementitious composite-filled double-skin steel tube (HPCFDST) beams strengthened longitudinally with various layers, lengths, and configurtion of CFRP sheets. The findings showed that, with increased CFRP layers, the moment capacity and flexural stiffness values of the retrofitted HPCFDST beams have significantly improved. For an instant, the moment capacity of HPCFDST beams improved by approximately 28.5% and 32.6% when they were wrapped partially along 100% with two and three layers, respectively, compared to the control beam. Moreover, the moment capacity of the HPCFDST beam using two partial layers of CFRP along 75% of its sufficient length was closed to the findings of the beam with two full CFRP layers. For energy absorption, the results showed a vast disparity. Only the two layers with a 100% full length and partial wrapping showed increasing performance over the control. Furthermore, the typical failure mode of HPCFDST beams was observed to be local buckling at the top surface near the point of loading and CFRP rapture at the bottom of effect length.

## 1. Introduction

Concrete-filled steel tubes (CFST) are known as structural composite members, wherein the voids of steel tubes are filled with concrete to enhance stiffness and load-bearing capacity [1]. The inter-boundary stresses of structural members are key to engineering advanced composites. Thus, the inherent engineering properties of such materials have earned them the global application as a state-of-the-art technique in the construction industry [2,3]. In such a design pattern, structural members are combined, such as the case in concrete-filled steel tubes, to provide unprecedented structural properties as solutions to a focused situation [1]. Concrete-filled steel tubes have numerous benefits, several advantages over ordinary structural steel, and normal reinforced concrete applications. The infill of concrete is restrained by the steel tube, resulting in producing a tri-axial limit state of compression that upsurges, which causes a hike in the strain and strength capacities of the infill of concrete [4]. The concrete restrains the steel tubes from both global and local buckling. Consequently, the distortion capacity of a CFST compares favorably with those of hollow tubes [5]. Further, the composited strength of both the concrete and steel offers outstanding compressive axial load and stiffness capacity. This makes the composite highly suitable for compressive structural applications, such as in columns [6]. Concrete-filled steel tubes is also permitted a fast construction since the steel tube abolishes the formwork and reinforcement related to concrete infill and reinforced concrete construction, which is quickly filled and formed [1]. The constituents of HPC contribute most efficiently to different structural requirements including strength, toughness, energy absorption capacity, durability, corrosion resistance, and damage tolerance when subjected to large deformations in structural members [7].

It is reported that, in cases of long-span structural members that require restriction in cross-sectional dimensions and that experience seismic forces, quivers, and vibrations, for such a situation, structural steel-filled concrete offers the best solution [8]. It combines the advantages of the concrete and steel such as the optimized cross-sectional dimensions, high strength, stability, and toughness [3,9]. The flexural behaviors of these concrete-filled steel tube (CFST) composite beams have been increasingly researched by engineers in the last two decades to enable the common use of (CFST) in structural projects [10]. Usually, aside from the fact that the beams, when compared to their hollow steel tube counterpart beams, display a high self-weight in return, they exhibit specific strengths, such as higher ductility, moment capacity, and stiffness. Concrete filled into the tube may significantly reduce or protect local buckling which happens mostly in the hollow tubes’ compressed zones [11]. Strengthening is, however, needed in the CFST flexural members because of deterioration due to the natural and environmental factors and possible upgrade of a structural member to accommodate additional loads [12]. Project funding, often times, are significantly raised as a result of the strengthening practices for the present steel and composite structural parts by replacement and addition of new steel elements, because these repair works are time and capital intensive [13]. In recent years, engineers have made utilization of carbon fiber-reinforced polymer (CFRP) schemes for steel sections’ strengthening [12,13,14]. These schemes are suitable for resisting harsh weathers and possess high strength and elasticity compared with its weight ratio higher than those of the steel [15].

**Figure 1:**
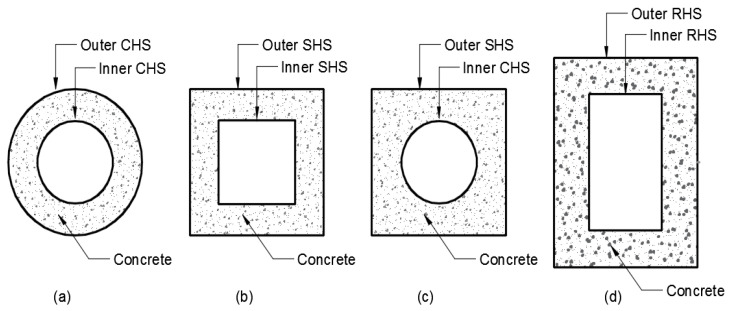
Cross-sections of double skin steel tube [20] (**a**) Circular hollow section (CHS) inner and outer, (**b**) Squre hollow section (SHS) inner and outer, (**c**) Circular hollow section (CHS) inner and squre hollow section (SHS) outer), and (**d**) Rectangular hollow sections (RHS) inner and outer.

The use of CFRP in steel members has been investigated in previous studies. The adoption of different retrofitting techniques, application of loads, and behavioral cohesiveness across the CFRP and steel boundary of composited elements have equally been critically analyzed [6]. Generally, the strengthening of CFST flexural members with CFRP sheets of one direction using the full strengthening sheet of their cross-section has been examined previously in various studies [5]. Carbon fiber-reinforced polymer (CFRP) sheets that can effectively resist the high-tension stress are utilized to mainly upgrade the capacities of both the simply wrapped steel and concrete-steel I-beams when equally laid along their tension flange [16]. In addition, it could be difficult to achieve an adequately strengthened cross-section by reducing the quantity of the CFRP in a partially retrofitted CFST beam. This is so because loads are applied on the top flanges of beams, typically, when used as girders for bridges [17]. Employing the CFRP schemes could thus be considered as an appropriate solution when following the same strengthening concept of steel-beam as the simply supported CFST beams are strengthened from the bottom flanges [18]. Another composite construction innovation is known as the concrete-filled double-skin steel tubes (CFDST). Although the CFDST members display almost all the strengths of the traditional CFST members, they exhibit a lighter in weight, higher bending stiffness, and better cyclic performance [19]. Two concentric steel tubes with concrete sandwiched within them are what embedded in this innovative composite construction. The steel tubes can be circular hollow sections (CHS), square hollow sections (SHS), or rectangular hollow sections (RHS) [20] which eventually lead to several possible combinations as shown in Figure 1.

Yu et al. [21] have studied the flexural behavior of hybrid FRP concrete steel double-skin tubular beam (DSTB). The specimens exhibited high ductility performance and high shear resistance. The authors concluded that the flexural response of a DSTB, including the flexural stiffness, the ultimate load, and cracking can be substantially improved by shifting the inner steel tube toward the tension region or providing FRP bars as additional longitudinal reinforcement. Uenaka and Kitoh [22] have investigated the mechanical behavior of a concrete-filled double skin circular (CFDST) hollow beam. The results presented that the failure mechanisms of the tested beams were governed by the parameter di/do (diameter of inner tube over a diameter of the outer tube) where the beams with highest di/do, experienced filled concrete crushing in the early stage of loading. Moreover, Idris and Ozbakkloglu [23] have studied the effects of varying the inner diameter of steel tube and the use of mechanical connectors on the steel tube on the flexural behavior of hybrid circular double-skin tubular beams under static load. The results showed high inelastic deformation and minimal strength degradations. The higher inner diameter decreased the slip between the concrete and the steel tube of DSTB as well as the mechanical connectors. Therefore, the flexural capacity of the tested beams was improved significantly. Concrete-filled, double-skin steel tube (CFDST) beams using CFRP sheets and high-performance fiber-reinforced cementitious composite have never been investigated to date. This research, therefore, aims to study the effectiveness of employing the unidirectional CFRP sheets in the strengthening of the high-strength rectangular double-skin steel tube beams filled with high-performance reinforced fiber composite concrete under four-point bending. The effects of various parameters, such as configuration, multiple CFRP layers, and varied wrapping lengths of CFRP in high-performance fiber-reinforced cementitious filled-double skin steel tube (HPCFDST) beams, were investigated.

Previous laboratory works have been studied the bending performance of CFST [16,17,18,19,20,21,22,23,24]. The major findings provided an overview of the CFST bending behavior; nevertheless, the investigational campaigns were limited, and, consequently, it is tough to examine the full-scale specimens to determine significant parameters of the design aspect [17]. Further empirical studies and analytical finite element methods (FEM) would buffer and complement the previous findings. The analytical FEM would offer a great opportunity to investigate parameters such as sizes, geometry, and structural arrangements that are difficult to replicate in actual experimental exercises [18]. Several studies have engaged macro-element and fiber-based models to analyze the CFST column and beam [25]. The use of fiber-reinforced polymer (FRP) composites in the fabrication of novel high-performance composite elements, such as beams and columns, in the shape of CFFTs, has been widely become common and tested under flexural loads [26,27].

As proceedings from past studies on CFFTs, a novel composite design of steel tubes with embedded CFRP tubes filled with concrete has gained prominent research interest [1]. The double-skin tubular columns and beams (i.e., DSTCs and DSTBs) depend on a similar CFRP tube confinement apparatus that is shown in CFFTs. In that case, the integration of the benefits of the three essential materials can be harnessed to display good structural performance. Several laboratory investigations have been considered on the composite components [1,18,27,28,29,30]. However, there are some gaps in the existing studies, which include the compatibility of infilled concrete with the skin section materials. In most cases, concrete was much weaker than the skin sections, therefore either the failure happened in concrete, whereas the skin sections were stressed at moderate to a low level, which would be an uneconomical solution. Therefore, this study used high-strength fiber-reinforced concrete to optimize the load-carrying capacity of the steel section and infilled concrete.

The main findings of these investigations were the the performance features provided by the CFFT composites subjected to different loading conditions. Whereas, the most significant role of such design systems is its efficacy in result verifications. For the DSTC and DSTB application, the composite behavioral features between concrete infill and double-skin steel tube filled with ultra-high cementitious materials integrated with CFRP sheets are yet to be studied. Thus, the aim of this study was to experimentally investigate the flexural behavior of a double-skin steel tube filled with high-performance concrete and strengthened with CFRP sheets and to compare the results with an analytical study using equations presented by four different codes.

## 2. Materials and Method

### 2.1. Material Characteristic

Two different cross-sections of rectangular hollow steel tubes were utilized to fabricate the test specimens. The steel properties were given by the supplier as shown in Table 1. Three types of mix design, as shown in Table 2, were proposed with and without the addition of steel fiber to achieve high compressive strength. The water-to-binder ratio was selected to maintain the same workability (slump flow) of all mixes; therefore, it was adjusted accordingly. The fresh properties were determined and tested elsewhere by Nikbakht et al. [31]. The mix design was utilized as a filled mix in the double-skin steel tube beam specimens. The CFRP sheets of SikaWrap- 231C were utilized as strengthening the material. Sikadur-330 was utilized as an adhesive (epoxy) material to attach the CFRP sheets with the steel tubes and the duplicated sheets together. This adhesive material is a mix of two parts of resin (A) and hardener (B) at a ratio of 4:1 by weight. The properties of CFRP and epoxy are shown in Table 3 as given by the manufacturer.

Ordinary Portland cement, type-1, was obtained from Lafarge, Malaysia; its specific gravity was 3.5 and conforming to the ASTM C150. Fly ash satisfying ASTM C618 specifications was acquired from a coal fire power station in Manjung, Malaysia. Mean particle size (D50), of fly ash, was determined as 24 microns, and for silica fume, it was 0.16 microns. The BET surface area of fly ash was determined as 0.994 m^2^/g, and loose bulk density of fly ash was 860 kg/m^3^. Well-graded coarse aggregate was used with size variation from 4.75 mm to 20 mm and fineness modulus 7.23. The water absorption and specific gravity for coarse aggregate were 0.44% and 2.5%, respectively. River sand was used as a fine aggregate with fineness modulus 2.2, water absorption of 1%, and specific gravity 2.55. Superplasticizer Sika Viscocrete 2044 confirming the specifications of ASTM C494 was used with drinkable water. Copper coated 20 mm long was used, and 0.3 mm in diameter and 25 mm length steel fibers with 2300 MPa were used [32].

### 2.2. Test Specimens

**Figure 2:**
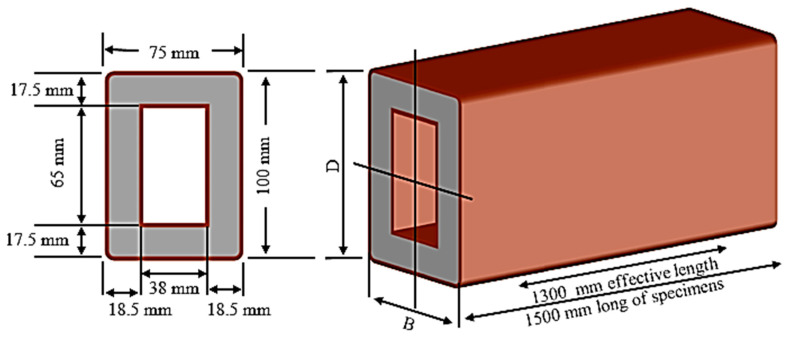
High-performance fiber-reinforced cementitious filled double-skin steel tube (HPCFDST) beamcross-section.

Steel tube beams were utilized with a cross-section established at 75 mm in width, 100 mm in height, and 1500 mm long for the outer steel measurement and 38 mm in width, 65 mm in height, and 1500 mm long for the inner steel measurement. The steel tube thickness was 2.3 mm. However, both steel tubes consolidated to deliver double-skin steel tube beams. Figure 2 demonstrates the cross-section of CFDST.

**Figure 3:**
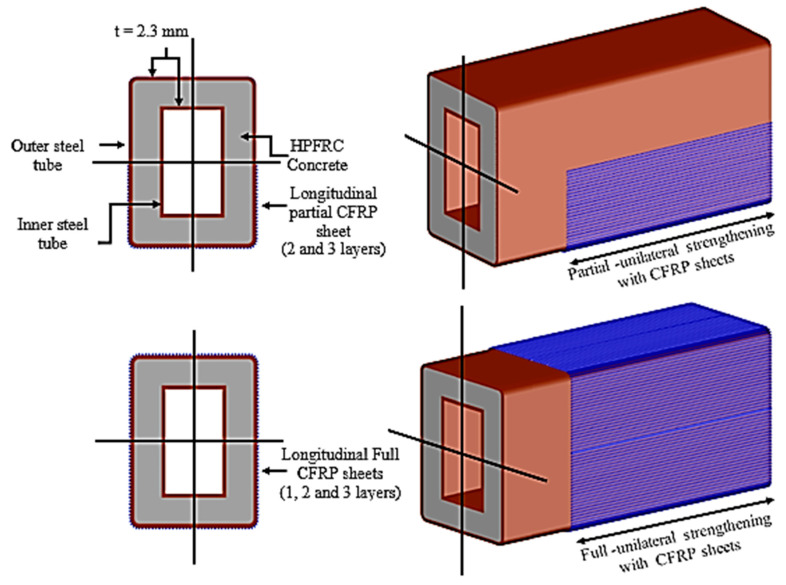
CFRP wrapping sheets: (**a**) partial wrapping scheme for HPCFDST beams, (**b**) full wrapping scheme for HPCFDST beams.

Eight rectangular specimens of high-performance fiber-reinforced cementitious filled double-skin steel tube (HPCFDST) beams were subjected to four-point bending flexural load. The strengthening of the beams was done by attaching the CFRP schemes along the lower half of the member’s cross-sections, which recognized as the partial-unilateral CFRP strengthening scheme; the other type of strengthening was the fully wrapping kind. Details of the labeling beams are shown in Figure 3 and presented in Table 4. B refers to the cross-sections of the beams, while the control specimens’ sample is C. The partial wrapping in one direction is represented by the numbers and letter P100, P75, and P50 which have their reputation as a “U-shaped” scheme (P) applied along with a varying percentage of the beams’ effective lengths (Le): 100%, 75%, and 50%, respectively. The total number of CFRP layers are represented by the last number and letter (for instance, 3L = three layers). The full lengths of three rectangular beams were fully strengthened with the use of different CFRP layers parallel to the beam such as (B-F100-2L); it is known as the “full” scheme (F). Partial wrapping was used for the other six beams with varying lengths and layers. The full and partial-unilateral wrapping scheme, represented by the effective length of specimens, which were 1300 mm from support to support, moreover, the depth to thickness (D/t) ratios was 43.

### 2.3. Preparation of Specimens

Prior to beam specimens test, for each mix, three cubes of 100 × 100 × 100 mm were cast and tested under compression at 7, 14, and 28 days following the procedures of ASTM C39-12 [34]. The average compressive strength was reported, and the optimal mix was then chosen to cast the beam specimens.

**Figure 4:**
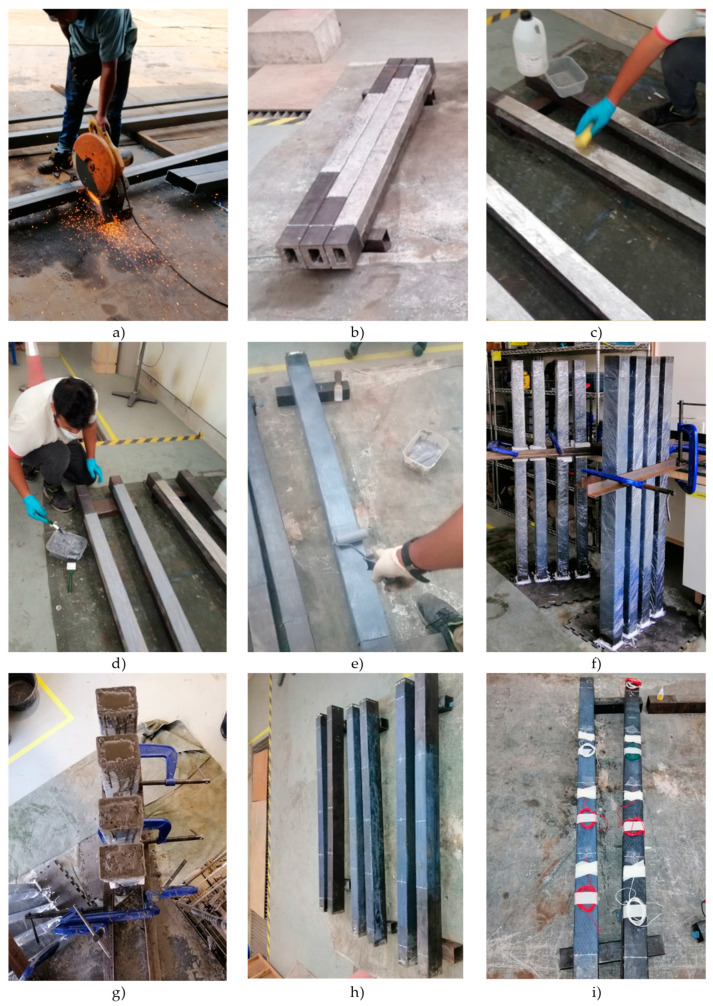
(**a**) Cutting and Fabrication of beams; (**b**) grinding of beams; (**c**) cleaning of beams by acetone; (**d**) applying of epoxy; (**e**) attaching CFRP; (**f**) Setting-up of beams; (**g**) casting concrete; (**h**) marking place of stain gauges; (**i**) attaching strain gauges.

The electrical saw machine was used to cut all steel tube beams to 1500 mm by the supplier, as shown in Figure 4a. Afterward, double skin steel tubes fabrication was done on all specimens in the workshop by welding small steel tube cross-section inside the big steel tube beams. A low-speed electrical grinder was then utilized with sandpaper (24 grit) to roughen the pre-marked zone only from the carbon layer, this was also to ensure that there was no rust, paint and other undesirable material on the outside of the steel tube and as a result of grinding these tubes with this type and size of sandpaper, the roughness of the surface increased, and it interlocked the CFRP with the surface of the steel tube beam as shown in Figure 4b. Acetone liquid was used to clear the surface of the steel of the grinding dust and dirt. This is illustrated in Figure 4c. Aside from the fact that the surface of the steel tube was clean, the adhesive material, Sikadur-303, (epoxy) was mixed according to the manufacturer’s instructions by 4:1, and the sticky materials were applied into the steel tube surface immediately as shown in Figure 4d. Then, simultaneously and in a way that is parallel to the beam’s direction, the first and second layer of CFRP was attached instantly around the steel tube beams, respectively. The air void between the CFRP and steel tube beam was removed by a unique ribbed roller to ensure that the thickness of the adhesive material was uniformly distributed along the effective length of the beam, as shown in Figure 4e. The specimen was cured under room temperature, and all specimens were cover by a roller-plastic to keep it clean from concrete casting. Next, the steel tube beam was set upstanding and supported by G-clamp for the more effortless casting of the HPC inside the beam. A silicon pad was glued to the bottom part of the steel tube beam, which temporarily secured it to avoid water leak during the process of placing the HPC as shown in Figure 4f. One concrete batch was used to cast two beams according to the cross-section type and concrete mixer capacities as shown in Figure 4g. In continuation, the end of strain gauges was soldered with wire before being positioned in the proper location after marking it with a pen, as shown in Figure 4h. A strain gauge resistance of 120 Ω was attached and glued using Araldite and 3-second glue as shown in Figure 4i. Once the sample was ready, the beam stayed placed in its correct position inside the UTM machine as in Figure 4h. The strain gauge was then tested with a multi-meter to make sure it has not broken before the test. After that, the load was applied at the rate of 0.5 kN/min on the beam, and the specimen was deflected until a maximum force of 450 kN was reached, and at this point, a rupture occurred in the outer surface of the test specimen.

### 2.4. Instrumentations and Testing Setup

The composite beams (infilled concrete in double-skin steel tubes) were designed to behave as a flexural member. Therefore to predict the failure mechanism, the test setup was designed so that the maximum stress should be induced within the middle–third span. Hence, the strain gauges were installed in such a manner that the dominating mode of strain could be identified via testing, and data could be used for flexural analysis.

**Figure 5:**
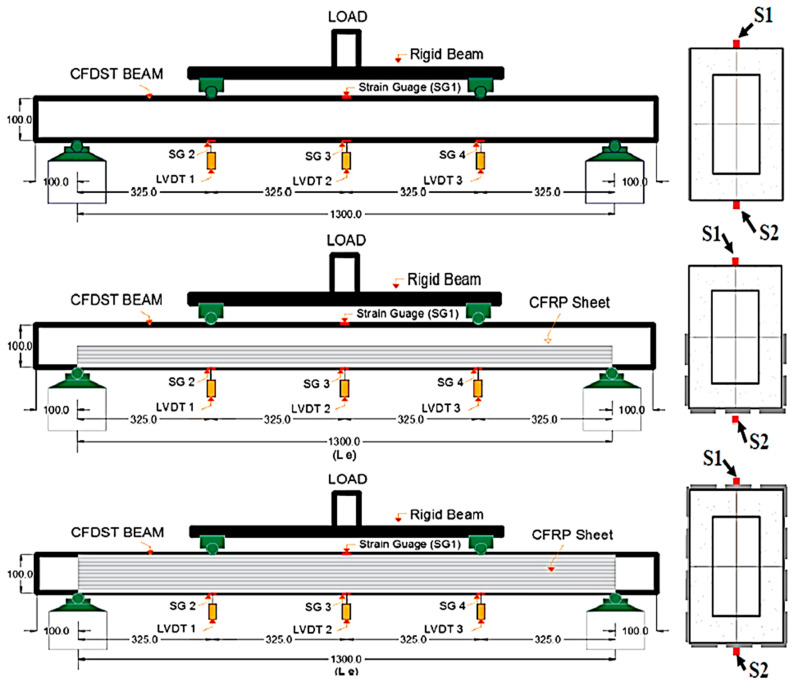
Setup of the test of HPCFDST beams strengthening with CFRP scheme (all units in mm).

The four-point bending tests were utilized in this study to locate the maximum load capacity of HPCFDST beams. A dynamic machine with a limit capacity of 500 kN was used. Three LVDTs were utilized to measure the displacement. The strain gauge was used to study the tensile stress at a different location of the beam. The positioning of the LVDT is based on the critical area of deflection. More so, it was at the bottom of the beam because the maximum deflection is expected to be at the bottom and center of the beam. A total of four strain gauges was utilized in the investigations. One strain gauge was situated on the top of the beam and the other three at the bottom of the beam, as shown in Figure 5.

## 3. Test Results and Discussion

### 3.1. Compressive Strength of HPFRCC

**Figure 6:**
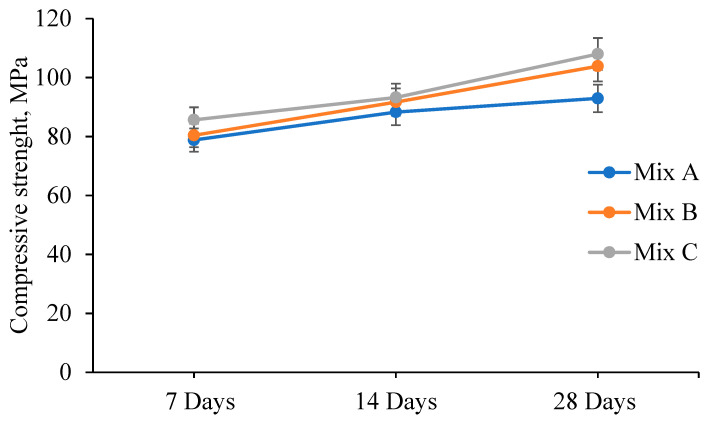
Trial mix of the compressive strength results.

The concrete mix design was built to help to achieve a compressive strength. The compressive strength of the trial mixtures results is shown in Figure 6. The average result of the compression strength of the concrete cubes for Mix A, B, and C at 28 days is 92.96, 103.89, and 108.03 MPa, respectively. Mix C shows higher compressive strength due to the presence of steel fiber. Thus, mix C was used to cast the beams throughout the test.

### 3.2. Results of HPCFDDST Beam

#### 3.2.1. Moment Carrying Capacity

**Figure 7:**
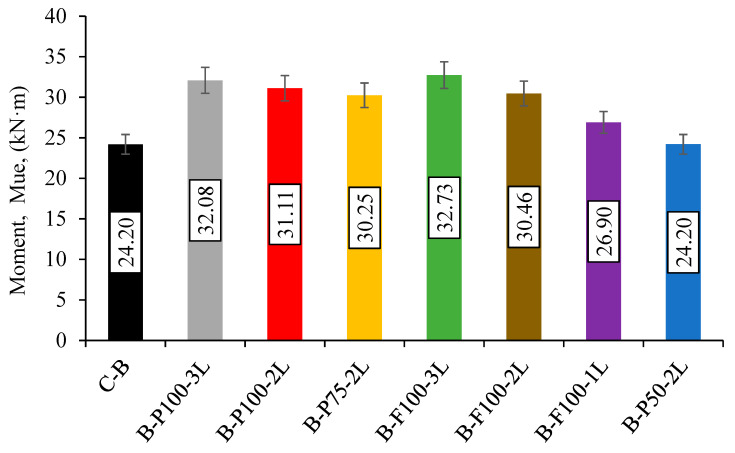
Ultimate moment capacity (M_ue_) of beams.

All Strengthening specimens’ ultimate moment capacity (M_ue_) either ruptured or delaminated when the limit of their CFRP sheets was reached. The M_ue_ ratio reached by the retrofitted sample to the maximum control sample capacity was used to calculate the load improvement ratio (LIR). The LIR has been determined based on the control sample (B-C) capacity for the HPCFDS samples strengthened with CFRP. Table 5 shows the sample values of M_ue_ and LIR, while in Figure 7 is a comparison of the values of M_ue_ for the tested HPCFDST samples. Achieving an LIR of about 1.35, the moment capacity of the sample with the fully retrofitting sheets (B-F100-3L) was found to be 32.7 kN·m; this was the highest of the LIR values with full and partial specimen schemes. Generally, the increase in the moment capacity of the strengthening HPCFDST beams was as a result of a rise in the number of layers of the CFRP. The moment, Mu value of the B-P100-2L sample (2 CFRP layers) was 31.1 kN·m, which later improved to 32.1 kN·m as a result of using three CFRP layers (B-P100-3L) an LIR of 1.33 was thus achieved. The sample’s capacity of (B-P75-2L) was raised to + 25% more than the control samples’ average capacity because of using two layers of CFRP along 75% of its effective length which was extremely close to the result obtained for the B-F100-2L specimen (+26%) as in either specimen, no CFRP delamination failure occurred. Once they reached their maximum tensile strength, the CFRP for both samples ruptured from the bottom mid-span zone. Meanwhile, the HPCFDST beam’s capacity could not be enhanced by the 50% strengthened length due to the earlier CFRP delamination failure. However, due to the fact that the CFRP schemes were positioned along the half-length of the beams and the bonding strength between the CFRP and steel tube in this zone was lower than the peeling stress, the roughness of the steel structure was not sufficient to prevent the failure of the delamination; this finding is in line with other studies [29]. The effect of the roughness of the steel surface also corresponded to the findings of previous studies, although not very significant in this case, and the studies in focus concluded that the roughness of the surface of steel and the characteristics of the adhesive (epoxy) material would determine to increase the mechanical bond interaction as the strength of the bonding between the two adherent parts can improve by increasing the roughness of the steel surface [33].

#### 3.2.2. Relationship of Moment–Curvature Responses

**Figure 8:**
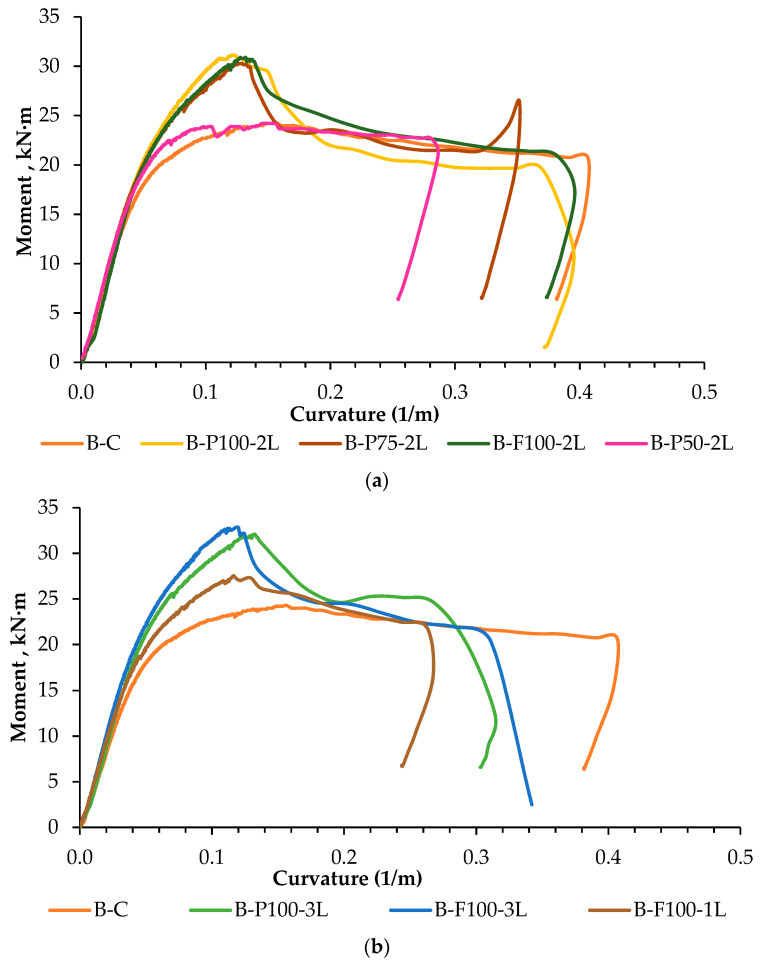
8 relationship of HPCFDST beams (**a**) control beam, partial, and full strengthening with 2 layers of CFRP, and (**b**) control beam, partial (3 layers), and fully strengthening (1 and 3 layers) with various CFRP layers.

Figure 8 shows the relationship of moment–curvature of HPCFDST beams laminated with the CFRP sheets. The beams displayed the elastic behavior at the beginning of load application until a curvature (1/m) of approximately 0.1 was reached. Afterward, the moment–curvature relationship showed a plateau in the graph, which indicates an inelastic behavior of the beams. The laminated samples behaved inelastically when the CFRP sheets started delaminated or ruptured. Figure 8a showed the relationship of moment–curvature of the reference beam (B-C), and the beams partially and fully laminated using two layers of CFRP sheets. The beams laminated to 100%, and 75% of span length (B-P100-2L, B-F100-2L, B-P75-2L) showed about 30% enhancement in the maximum moment in comparison with the reference beam whereas the beam laminated to 50% of the span length (B-P50-2l) achieved almost the similar value of the maximum moment. Figure 8b shows the relationship of moment–curvature of the reference beam and the fully and partially wrapped beams to 100% span length using one layer (B-F100-1L) and three layers of CFRP sheets (B-P100-3L and B-F100-3L). It is observed that the beams wrapped with three layers of CFRP achieved about 40% higher ultimate moment than the control beam B–C, whereas the fully wrapped beam laminated to full-span length using one layer of CFRP sheet showed and enhancement in the maximum moment of about 10%. By comparing the effects of full wrapping and partial wrapping of the beams, it was not notice any significant improvement in the maximum moment capacity. It was observed that lamination to 50% span length caused a considerable reduction in the maximum moment capacity of the beams as compared to lamination to 75% and 100% of the span length. From these results, it can be concluded that the partially wrapped beam using two layers of CFRP sheets and laminated to 75% span length (B-P75-2L) is considered as the optimum design that carried 30% higher moment than the reference beam (B-C) and achieved an ultimate curvature (1/m) of 0.32.

**Figure 9:**
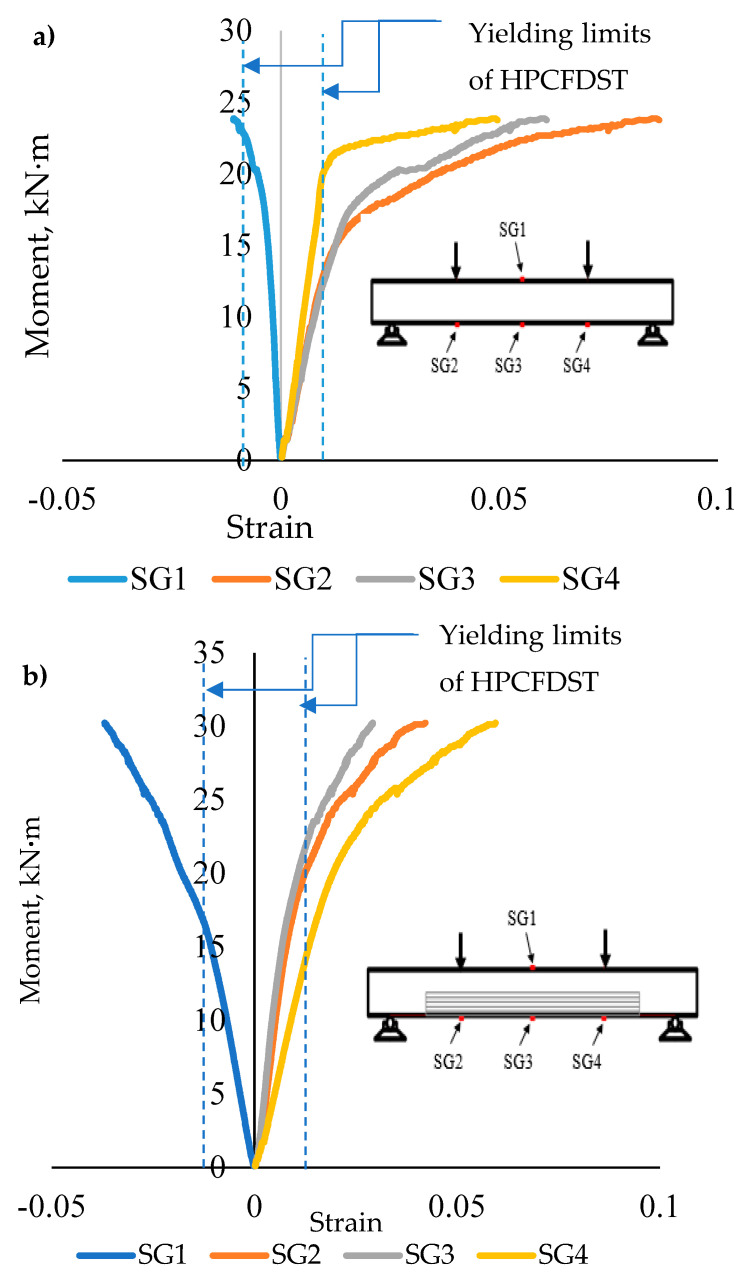
Relationship between moment and strain for (**a**) B–C and (**b**) B-P75-2L specimens.

The relationships between moment versus strain for the control specimen (B-C) and (B-P75-2L) is shown in Figure 9, the tensile strain on the tension zone of the simply supported beams augmented progressively along the half-span to the support point, as detected by electrical-strain gauges SG4 and SG2 as shown in Figure 9a.

#### 3.2.3. Energy Absorption

**Figure 10:**
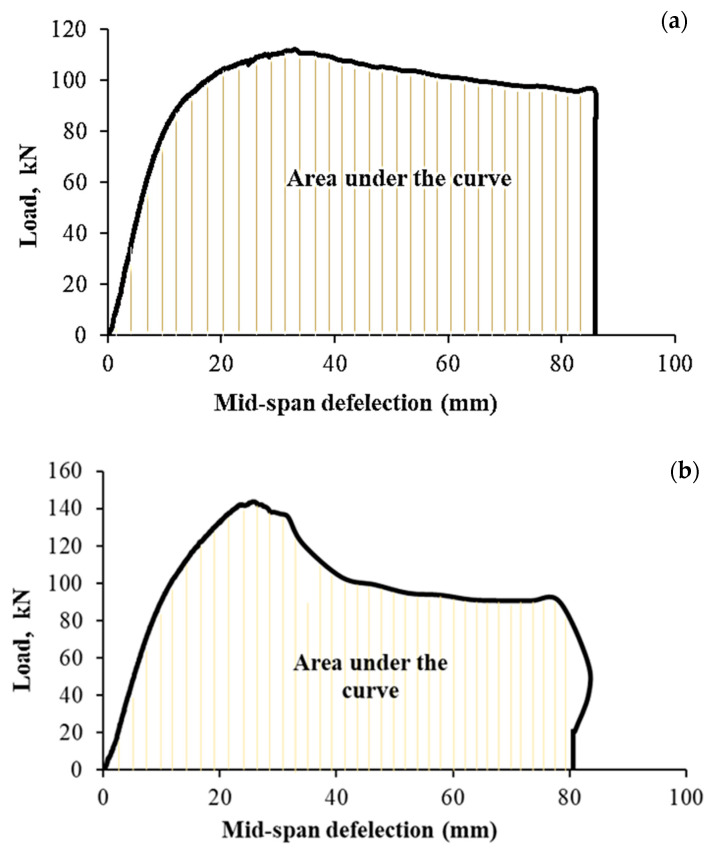
Characteristic load versus midspan deflection curves for assessing the energy absorption, (**a**) control specimen (B-C) and (**b**) strengthened specimen (B-P100-2L).

**Figure 11:**
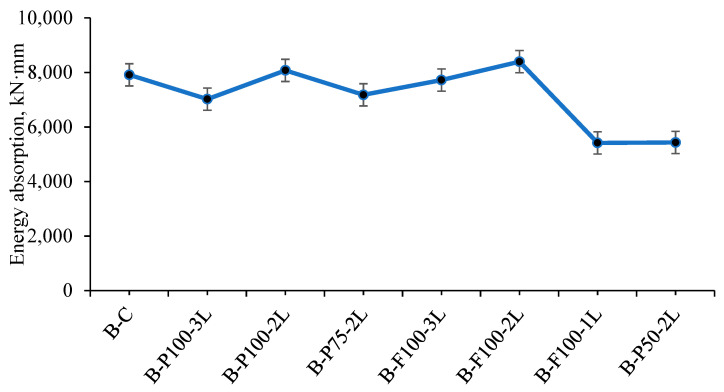
The value of energy absorption (EA) capacity for all specimens.

The energy absorption capabilities of seven totally different specimens (B-P100-2L, B-F100-2L, B-F100-1L, B-P50-2L, B-P100-3L, and B-F100-3L) have been studied in comparison with the control beam (C-B). The energy absorption capability can be determined by calculating the area under the envelope (curve) of load against deflection at mid-span of all the tested specimens. The load-deformation curve for two selected specimens, control beam (B-C) and strengthened specimen (B-P100-2L), from which areas were evaluated, as shown in Figure 10a,b. Based on this and as shown in Figure 11, it can be expressed that the absorption of energy of the B-P100-2L and B-F100-2L specimens has shown the most increase, among all tested samples, from 7912.8 kN·mm, recorded for control beam, to 8076.31 and 8396.1 kN·mm, correspondingly. It is noteworthy, however, that the subject of the specimen to delamination and rupture primarily at the early loading stage can go a long way in affecting their performance. This has been the case for the B-P50-2L and B-F100-1L specimens. They have the lowest energy absorption capacity values of 5416.3 and 5431.14 kN·mm, respectively. Additionally, it can be seen that the B-P100-3L and B-F100-3L specimens have slightly lower energy absorption values than the control beam. This is due to the formation of three new layers of CFRP at the addition of high-performing fibers to the cementitious concrete, leading to an increase in stiffness and a corresponding decrease in ductility. As a conclusion, it is recommended to use two fully or partially wrapping layers for better efficiency of the beam.

#### 3.2.4. Flexural Stiffness

**Figure 12:**
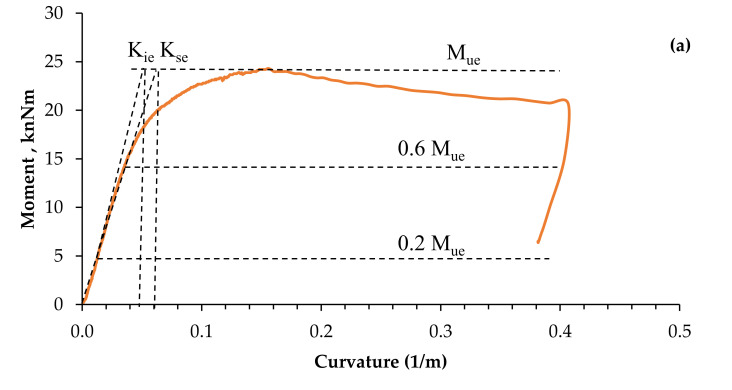

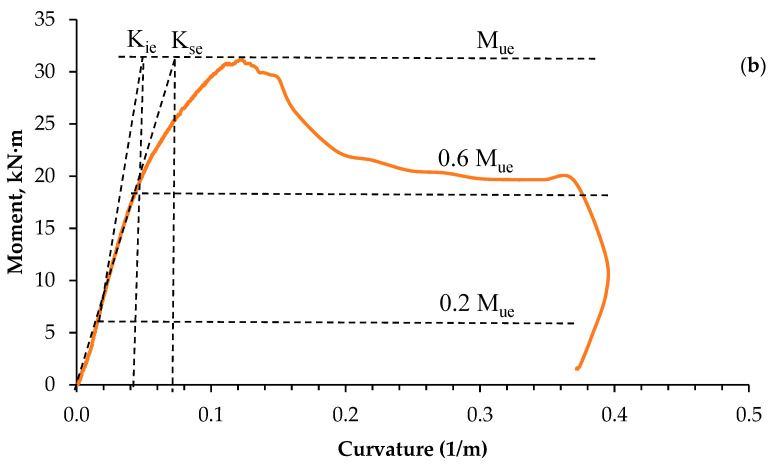
Typical relationships of bending moment–curvature: (**a**) reference specimen (B-C) and (**b**) integrated specimen (B-P100-2L).

**Figure 13:**
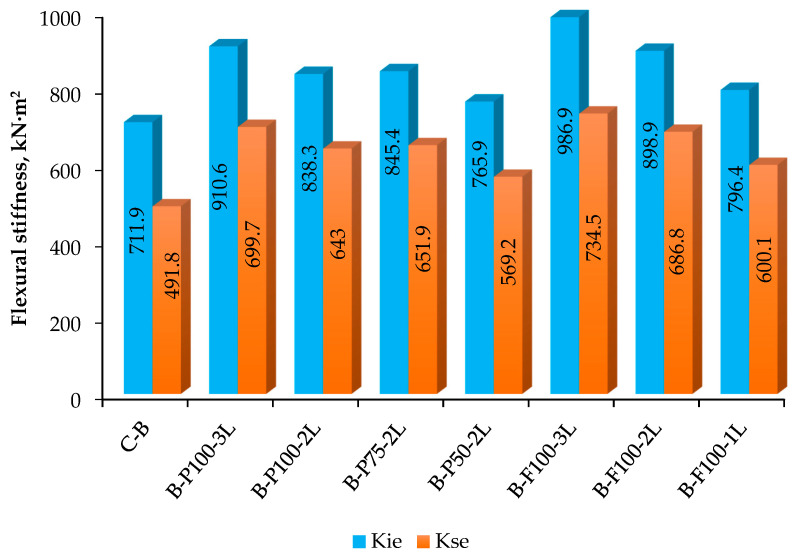
Flexural stiffness (K_ie_ and K_se_) of HPCFDST specimens.

It is found that the impact of the addition of CFRP layers on the stiffness of the flexural of HFCFDST beams. Specimens B-C and B-P100-2L were chosen as typical examples for illustrating changes in flexural stiffness, determined from moment–curvature relation, as shown in Figure 12. The moment–curvature curve is applicable in the determination of the serviceability-level section flexural stiffness (K_se_) and the early section flexural stiffness (K_ie_) for every specimen. The K_se_ and K_ie_ are the secant stiffness; that are corresponding to the serviceability-level moment of 0.6 M_ue_ and equivalent to the moment of 0.2 M_ue_, respectively [30,35]. The two specimens initially exhibited elastic behavior before been pulled down gradually to an inelastic condition where eventual moment capacity (M_ue_) was attained. Table 5 presents the experimental results of K_se_ and _Kie_ resulting from the test done on HPCFDST samples. This can be further seen graphically in Figure 13. From the two results, we observed that both K_se_ and K_ie_ values augmented linearly with an increase in CFRP layers; the K_se_ and K_ie_ values for specimen B–C were elevated from the initial values of 491.8 kN·m^2^ and 711.9 kN·m^2^, in the absence of CFRP sheets, to 699.7 kN·m^2^ and 910.6 kN·m^2^, when reinforced with 3 CFRP sheets. This indicates the increment in the stiffness quality of about 27.9% and 42.1% for K_ie_ and K_se_, respectively.

The actual flexural stiffness of CFST specimens has been estimated over a long time now through the conventional code methods; AIJ (1997) [36], EC4 (2004) [37], AISC (2010) [38], and BS5400 (1979) [39], and although, none of these methods have considered the role CFRP can play, the current experimental results indicated the enhancement efficiency that can be achieved in the flexural stiffness of HPCFDST beams by the addition of CFRP sheets. Therefore, in order to measure the effect of CFRP, this study recommends that the stiffness value of CFRP sheets should be obtained first and separate, and then added to the formulas of the conventional methods stated below:

1. AISC (2010)
  (1) $$K_{p}=E_{s}.I_{s}+C_{1}\;.\;E_{c}\;.I_{c}$$
  where, $C_{1}=0.6+0.2\;\left(A_{s}/\left(A_{s}+A_{c}\right)\right)\;\leq 0.9,E_{C}=4733\sqrt{f_{c}}$.
2. EC4 (2004)
  (2) $$K_{p}=E_{s}\;.\;I_{S}+0.6\;.\;E_{c}\;.\;I_{c},$$
  where, $E_{c}=9500\;\times \;{\left(f_{ck}+8\right)}^{\frac{1}{3}}\;,f_{Ck}=0.67f_{cu}$.
3. BS5400 (1979)
  (3) $$K_{p}=E_{s}\;.\;I_{S}+E_{c}\;.\;I_{c},$$
  where, $E_{C}=450\;\times \;f_{cu}$.
4. AIJ (1997)
  (4) $$K_{p}=E_{s}\;.\;I_{S}+0.2\;E_{c}\;.\;I_{c},$$
  where, $E_{c}=21000\;\times \;\sqrt{f_{c}/19.6}$.

Furthermore, the multi-CFRP sheets (the gluey sheets sandwiched were all taken as one sheet) have been represented with the CFRP patch technique as adopted by previous researchers [33]. The moment of inertia for every spot of the CFRP ($I_{CFRP.Patch})$ was estimated from the geometry of the integrating system via the overall CFRP patch thickness ($t_{CFRP,Patch})$. The thickness of every gluey layer attached to the strengthened specimens falls averagely between 0.8 and 1.0 mm after a series of measurements. The stiffness of CFRP ($K_{CFRP}$), the modulus of elasticity, and the total thickness of for each CFRP patch were assessed as follows:

(5) $$K_{cfrp}=E_{CFRP,Patch}\;\times I_{CFRP.Patch}$$

(6) $$t_{cfrp.\;patch}=\left(n\;\times \;t_{cfrp,\;sheet}\right)+\left(n-1\right)\;\times \;t_{ad}$$

(7) $$E_{cfrp.\;patch}=\left(n\;\times \;t_{cfrp,\;sheet}\;\times \;E_{cfrp.\;sheet}\right)+\left(n-1\right)\;\times \;t_{ad}$$

The summation of the entire flexural stiffness of the integrated HPCFDST samples $\left(K_{t}\right)$, projected by means of each of the techniques, and the CFRP patch stiffness ($K_{CFRP}$) was contrasted with the flexural stiffness at serviceability level and the original flexural stiffness ($K_{se}$ and $K_{ie}$) obtained from the experimental tests shown in Table 6 and Table 7, correspondingly. The standard deviation $\left(S_{d}\right)$, mean value (MV), and variation coefficient (COV) of the ratio of the anticipated value-to-test value for the variable design techniques were all reported in these tables. The outcomes in Table 7 display obviously that the techniques of BS5400, AISC, and EC4 were moderated in approximating the original flexural stiffness ($K_{t}/K_{ie}$) with MVs of around 0.606, 0.404, and 0.715, respectively. Also, Its COV ranges from 5.072 to 6.940. The AIJ technique, meanwhile, was the best predictor as it attained an MV of 0.404 and a COV of about 5.072. This was due to the fact that the AIJ technique implemented a lesser reduction factor of 0.2 to compute the concrete flexural stiffness in contrast with the other aforementioned techniques. In Table 5, the COV and MV of the ratio of $K_{t}/K_{se}$ for similar techniques were featured. It is clearly depicted in Table 5 that, at the serviceability level, the BS5400, EC4, and AISC techniques were highly moderated in the assessment of flexural stiffness, having, respectively, MVs of approximately 0.809, 0.540, and 0.956 and COVs from 8.340 to 10.063. The AIJ technique also exhibited significant values, marginally greater than the experimental test results, reaching an average value of 0.540 and a CV of 8.340.

#### 3.2.5. Failure Modes

**Figure 14:**
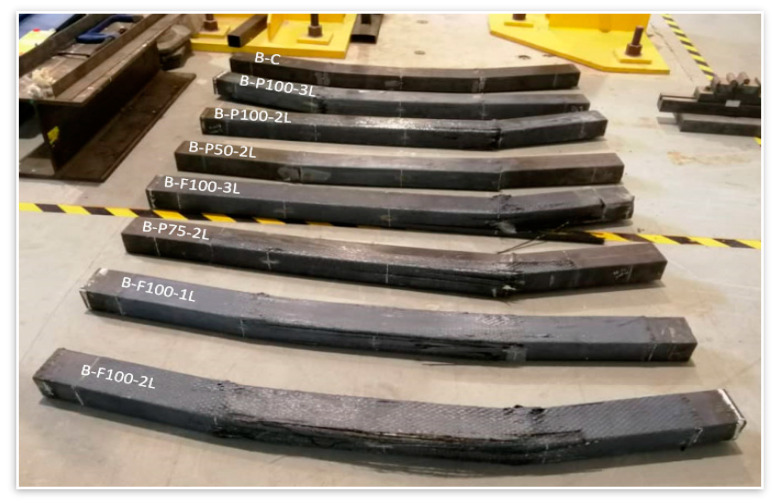
Failure modes of all tested HPCFDST samples.

**Figure 15:**
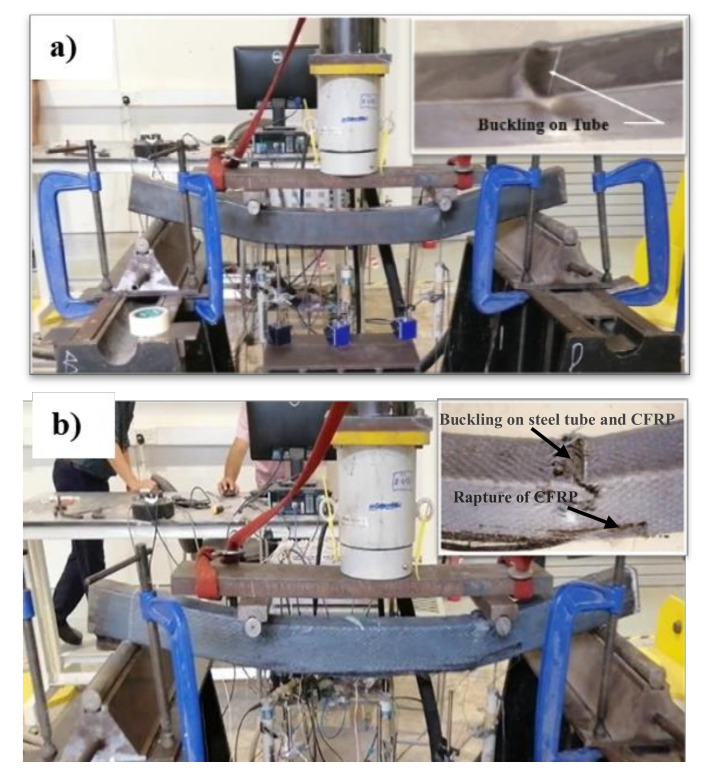
Buckling failure characteristic at the top surface of steel tubes (at the final loading phase); (**a**) B-C and (**b**) B-P100-3L.

At the ultimate loading stage, all specimens were overloaded to obtain more understanding of the behavior of HPCFDST specimens when integrated with the CFRP sheet at the ultimate load stage. Generally, even beyond the ultimate failure limit of the tested specimens, the loading test was continued. The modes of failure of the HPCFDST specimens are presented in Table 5. No change was noticed in the shapes of HPCFDST beams at the time of testing until they attained their ultimate strength capacities. For all tested specimens, the outward buckling failure (local) that was at the top surface of the steel (close to points of loading), were monitored and recorded together with CFRP wrapped schemes as shown in Figure 14. For instance, Figure 15a,b indicates a control sample (B-C) and wrapped sample (B-F100-3L), respectively. The main difference amidst non-strengthened and strengthened samples was that the wrapped specimens attained a higher maximum load capacity when the CFRP sheets ruptured. It then returned to virtually the same level as the non-strengthened until the deflection value of the outward local failure is nearly identical.

**Figure 16:**
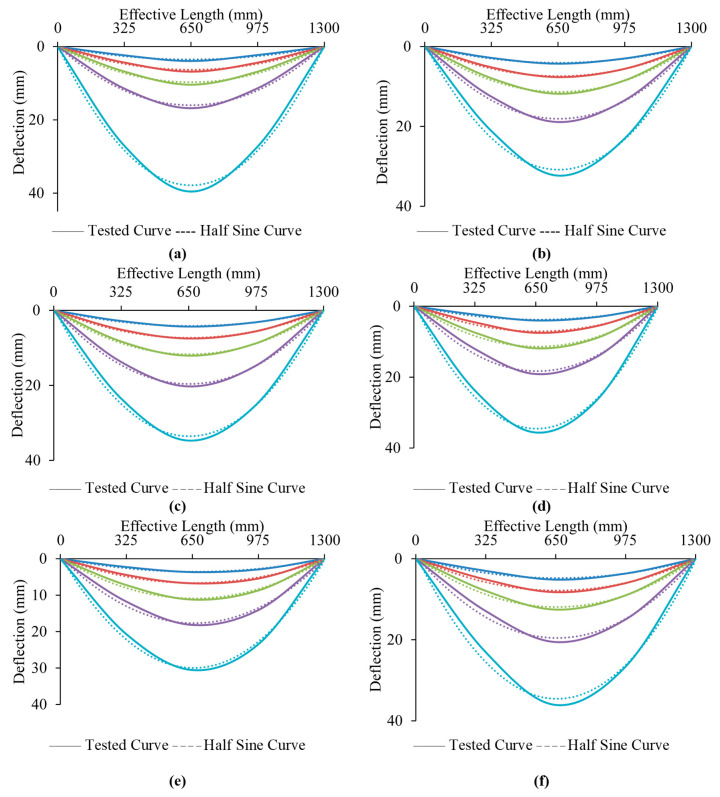
Deformation shapes in various loading phases: (**a**) B-C; (**b**) B-P100-3L; (**c**) B-P100-2L; (**d**) B-P75-2L; (**e**) B-F100-2L; (**f**) B-F100-2L.

**Figure 17:**
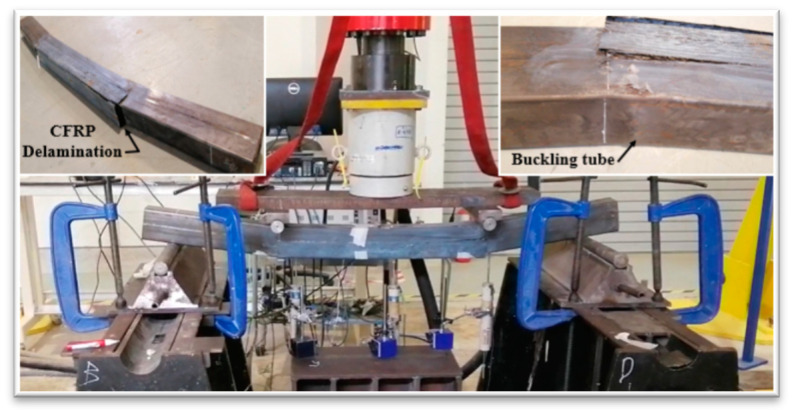
CFRP failure mode of (B-P50-2L) specimen.

Furthermore, for some selected samples, the curves illustrated the deflection behavior at various bending stages that are similar to the sine-curved shape, as displayed in Figure 16, while Figure 14 reflects the modes of failure of the samples. The pure-tension region first gave way to a small cracking sound in the CFRP (at the bottom center of the sample) when the wrapped samples got to about 85-90% of the maximum ultimate capacity. Similarly, other patterns of failure were observed for all samples. For these specimens, once the ultimate tensile strength is achieved, a rupture from the central portion is observed in their CFRP as a result of the high bending stress. The specimen with 50% strength lengths suffered delamination at the ends of the CFRP before their maximum strength was reached. This failure was accorded to a large volume of flaking stress that happened all over the bonding surfaces between the steel and CFRP layers as a result of the considerable bending stress at the peeling point which decreases gradually towards the end supports, discovering that delamination failure took place because the CFRP layers with 50% wrapped lengths were placed inside the zones where flaking stress is high as shown in Figure 17. However, the flaking stress might be overridden by the strength of bonding between the steel tubes and the CFRP layers for 75% wrapped lengths, hence, avoiding the occurrence of the failure mode of delamination or debonding.

It is concluded that the failure mode of (B-P75-2L, B-F100-2L, B-P100-3L, and B-P100-2L) beams is a one-sided failure. The reason for the single failure mode is associated with the incomplete compaction of the concrete within the beam’s specimens, thereby generating voids or opening within the beams. Similarly, there is the possibility of fiber agglomeration at one side of the beams that can make it stronger from the other sides. A steady CFRP rupture failure was noticed from the tension zone of beams under the point load in fully strengthening specimens B-F100-2L, B-100F-1L, and B-F100-3L. The no-slip failure between the steel tube and concrete core was seen in all specimens, as both ends of each specimen were visually checked and inspected during the tests.

#### 3.2.6. Summary of Study

This study focused on investigating the flexural performance of double-skinned steel beams filled with fiber-reinforced concrete (FRC). The significance of concrete-filled steel members was to design the very long flexural member with small cross-sectional dimensions. Through composite action, the cross-sectional load-carrying capacity could be enhanced. Most of the available research studies were focused on single and fully filled with concrete. Whereas, in the study, the double skin further enhanced the structural capacity and also reduced the amount of concrete to be filled. The FRC used was high strength and high performance in a nature that contributed better compatibility in stress transfer between steel sections and infilled concrete.

## 4. Conclusions

This study presents the flexural behavior of double-skin steel tube beams filled with fiber-reinforced cementitious composite and strengthened with CFRP sheets. The conclusion can be drawn as follows:

- The investigated of moment-carrying capacity results represent that the upsurge in the moment capacity of the strengthening HPCFDST beams was as a result of an increment in the number of sheets of the CFRP. For instance, the M_ue_ value of the B-P100-2 beam using two layers and B-P100-3L beams using three layers were raised to + 28.5% and 32.6%, respectively, compared to the control specimen. Moreover, the moment capacity of the B-P75-2L was raised to + 25% more than the control samples’ average capacity because of using two layers of the CFRP along 75% of its effective length which was extremely close to the result obtained for the B-F100-2L specimen (+ 26%) as in either specimen. Thus, it is better to use partial wrapping instead of full strengthening of CFRP for the purpose of cost-effectiveness;
- The moment–curvature responses of the HPCFDST beam strengthened with CFRP displayed elastic performance at the early loading stages and then plastic behavior with stiffness reducing progressively as exhibited by all specimens till the maximum moment capacity was achieved. The behavior of strengthened specimens after the CFRP rupture developed and became similar to the reference specimens until the ultimate strength capacity.
- Energy absorption can be concluded that the energy absorptivity of the specimens B-P100-2L and B-F100-2L showed the most increase, among all tested samples, from 7912.8 kN·mm, recorded for control beam, to 8076.31 and 8396.1 kN·mm, respectively. However, the remaining specimens had lower energy absorption values than the control beam. This was due to the formation of new three layers of CFRP at the addition of high-performing fibers to the cementitious concrete, leading to an increase in stiffness and a corresponding decrease in ductility. As a conclusion, it is recommended to use fully or partially wrapping of two layers for better efficiency of the beam.
- The flexural stiffness values increased linearly with an increase in CFRP layers; the Kie and Kse values for specimens B-C were elevated from the initial 711.9 and 491.8 kN·m^2^, in the absence of CFRP layers, to 910.6 kN·m^2^ and 699.7 kN·m^2^, when integrated with 3 CFRP sheets.
- The failure modes For all tested specimens displayed the outward buckling failure (local) that was at the top surface of the steel (close to points of loading). Similar patterns of failure were recorded for all samples. It is seen that when the ultimate tensile strength is achieved, a rupture from the central portion is observed in their CFRP as a result of the high bending stress. However, the specimen with 50% strength lengths (B-P50-2L) suffered delamination at the ends of the CFRP before their maximum strength was reached. Delamination failure took place because the CFRP layers with half wrapped extents were placed within the zones where flaking stress is high.

## Figures and Tables

**Table 1:** Hollow steel tube properties.

Cross-Section (mm)	Tube Location	Yield Strength, fy, (MPa)	Ultimate Strength, fc, (MPa)	Elongation (mm)
75 × 100 × 2.30	Outer	385	483	30
35 × 65 × 2.30	Inner	401	421	30

**Table 2:** Trial mix of high-performance composite concrete.

Ingredient (kg/m^3^)	Mixture A	Mixture B	Mixture C
Portland Cement	600	600	600
Fly Ash	390	450	390
Silica Fume	78.75	78.75	78.75
Water	194.25	194.25	194.25
River Sand	480	480	480
Superplasticizer	45.15	45.15	45.15
Steel Fiber	−	−	21.37
Quartz Sand	154.35	154.35	154.35
Water–Binder Ratio	0.18	0.17	0.18

**Table 3:** Carbon fiber-reinforced polymer (CFRP) and adhesive properties [33].

Item	Thickness (mm)	Maximum Tensile Strength (MPa)	Maximum Strain (%)	Elastic Modulus (GPa)
CFRP	0.13	4900	2.1	230
Epoxy (nominal)	2	30	0.9	4.5

**Table 4:** Labeling of HPCFDST beams strengthening with CFRP sheets.

No.	Labeling of Specimens	Type of Wrapping	Wrapped Length (% of span)	CFRP Layers
1	B-C	−	0	0
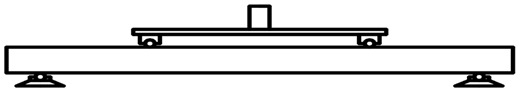				
2	B-P100-3L	Partial	100	3
3	B-P100-2L	Partial	100	2
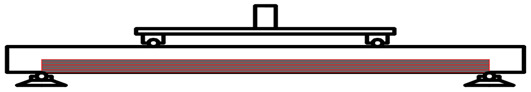				
4	B-P75-2L	Partial	75	2
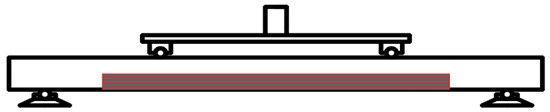				
5	B-P50-2L	Partial	50	2
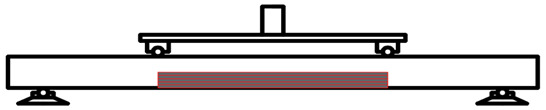				
6	B-F100-1L	Full	100	1
7	B-F100-2L			2
8	B-F100-3L			3
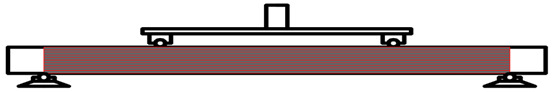				

**Table 5:** HPCFDST beams results.

Number	Specimen’s Designation	M_ue_ kN·m	LIR	EA kN·mm	K_ie_ kN·m^2^	K_se_ kN·m^2^	Method of Failure
1	C-B	24.2	−	7912.8	711.9	491.8	An outward buckling (local) at the steel tube’s top surface close to the points of loading.
2	B-P100-3L	32.1	1.33	7021.4	910.6	699.7	An outward buckling (local) at the steel tube’s top surface (one-sided failure).
3	B-P100-2L	31.1	1.29	8076.3	838.3	643.0	
4	B-P75-2L	30.2	1.25	7179.1	845.4	651.9	
5	B-P50-2L	24.2	−	5431.1	765.9	569.2	An outward buckling (local) at the steel tube’s top surface close to the points of loading.
6	B-F100-3L	32.7	1.35	7722.3	986.9	734.5	
7	B-F100-2L	30.7	1.26	8396.1	898.9	686.8	An outward buckling (local) at the steel tube’s top surface (one-sided failure).
8	B-F100-1L	26.9	1.11	5416.3	796.4	600.1	An outward buckling (local) at the steel tube’s top surface close to the points of loading.

**Table 6:** The comparative result among predicted total flexural stiffness (K_t_) and test results of K_ie_.

No.	Labeling of Specimens	Kie (kN·m^2^)	K_CRRP_ (kN·m^2^)	AISC (2010) Kt (kN·m^2^)	Kt/Kie	EC4 (2004) Kt (kN·m^2^)	Kt/Kie	BSS400 (1979) Kt (kN·m^2^)	Kt/Kie	AIJ (1997) Kt (kN·m^2^)	Kt/Kie
1	B-C	711.9	0	483.30	0.679	314.90	0.442	575.1	0.808	366.7	0.515
2	B-P100-3L	910.6	34.75	518.05	0.569	349.65	0.384	609.8	0.670	401.5	0.441
3	B-P100-2L	838.2	17.81	501.11	0.598	332.71	0.397	592.9	0.707	384.5	0.459
4	B-P75-2L	845.4	17.81	501.11	0.593	332.71	0.394	592.9	0.701	384.5	0.455
5	B-P50-2L	765.9	17.81	501.11	0.654	332.71	0.434	592.9	0.774	384.5	0.502
6	B-F100-3L	986,9	69.50	552.8	0.560	384.40	0.390	644.6	0.653	436.2	0.442
7	B-F100-2L	898.9	35.62	518.92	0.577	350.52	0.390	610.7	0.679	402.3	0.448
8	B-F100-1L	796.4	6.07	489.37	0.614	320.97	0.403	581.2	0.730	372.8	0.468
Average	−	−	−	−	0.606	−	0.404	−	0.715	−	0.466
Sd	−	−	−	−	0.039	−	0.021	−	0.050	−	0.026
COV (%)	−	−	−	−	6.464	−	5.072	−	6.940	−	5.594

**Table 7:** The comparative result among predicted total flexural stiffness (K_t_) and test results of K_se_.

No.	Labeling of Specimens	Kse (kN·m^2^)	KCRRP (kN·m^2^)	AISC (2010) Kt (kN·m^2^)	Kt/Kse	EC4 (2004) Kt (kN·m^2^)	Kt/Kse	BSS400 (1979) Kt (kN·m^2^)	Kt/Kse	AIJ (1997) Kt (kN·m^2^)	Kt/Kse
1	B-C	491.8	0.00	483.30	0.983	314.90	0.640	575.10	1.169	366.70	0.746
2	B-P100-3L	699.7	34.75	518.05	0.740	349.65	0.500	609.85	0.872	401.45	0.574
3	B-P100-2L	643.0	17.81	501.11	0.779	332.71	0.517	592.91	0.922	384.51	0.598
4	B-P75-2L	651.9	17.81	501.11	0.769	332.71	0.510	592.91	0.910	384.51	0.590
5	B-P50-2L	569.2	17.81	501.11	0.880	332.71	0.585	592.91	1.042	384.51	0.676
6	B-F100-3L	734.5	69.50	552.8	0.753	384.4	0.523	644.6	0.878	436.2	0.594
7	B-F100-2L	686.8	35.62	518.92	0.756	350.52	0.510	610.72	0.889	402.32	0.586
8	B-F100-1L	600.1	6.07	489.37	0.815	320.97	0.535	581.17	0.968	372.77	0.621
Average	−	−	−	−	0.809	−	0.540	−	0.956	−	0.623
Sd	−	−	−	−	0.078	−	0.045	−	0.096	−	0.055
COV (%)	−	−	−	−	9.626	−	8.340	−	10.063	−	8.826

## Notations

**Symbol**	**Description**	**Symbol**	**Description**
A_SO_	Outer steel tube area (mm^2^)	f_ck_	Characteristic of concrete strength (MPa)
A_SI_	Inner steel tube area (mm^2^)	f_y_	Yielding strength of steel tube (MPa)
A_HPC_	Area of high-performance concrete (mm^2^)	f_u_	Ultimate strength of steel (MPa)
B_S_	Width of outer steel tube (mm)	HPCFDST	High-performance fiber-reinforced cementitious filled-double skin steel tube
D_S_	Depth of outer steel tube (mm)	CFRP	Carbon fiber-reinforced polymer
b_S_	Width of inner steel tube (mm)	F	Full wrapping sheet
d_S_	Depth of inner steel tube (mm)	P	Partial wrapping sheet
B_C_	Width of outer concrete (mm)	I_SI_	Moment inertia of inner steel tube (mm^4^)
D_C_	Depth of outer concrete (mm)	I_SO_	Moment inertia of outer steel tube (mm^4^)
bc	Width of inner concrete (mm)	I_S_	Moment inertia of steel cross-section (inner and outer) (mm^4^)
d_C_	Depth of inner concrete (mm)	I_C_	Moment inertia of core HP concrete of cross-section (mm^4^)
EA	Energy adsorption (kN·mm)	L	CFRP layers attached on CFDST
EC	Modulus elasticity of concrete (GPa)	t	The thickness of the steel tube (mm)
E_SI_	Modulus elasticity of inner steel tube (GPa)	L_e_	Effective length (support to support) (mm)
ES_O_	Modulus elasticity of outer steel tube (GPa)	t_CFRP_, Scheme	Thickness of CFRP scheme (mm)
E_ST_	Total modulus elasticity of steel tube (GPa)	Kp	Flexural stiffness of HPCFDST Beam (kN·m^2^)
E_CFRP_, Scheme	CFRP scheme modulus of elasticity (GPa)	K_CFRP_	The flexural stiffness of CFRP sheet (kN·m^2^)
E_adm_	Modulus elasticity of adhesive martial (GPa)	K_t_	Total flexural stiffness of specimen (concrete, steel, and CFRP) (kN·m^2^)
f_cu_	Compressive strength of HP concrete cube	LIR	Load improvement ratio
M	Bending moment (kN·m)	K_ie_	Initial flexural stiffness of composite member (kN·m^2^)
M_ue_	Ultimate moment bending capacity	K_se_	Serviceability-level flexural stiffness of composite member (kN·m^2^)
B	Beam	φ COV	Curvature (1/m) Coefficient of variation
C	Control	S_d_ MV	Standard deviation Mean value or average
